# Refractory Hypoglycemia and Seizures as the Initial Presenting Manifestation of Empty Sella Syndrome

**DOI:** 10.7759/cureus.2803

**Published:** 2018-06-13

**Authors:** Vinoth K Sethuraman, Stalin Viswanathan, Rajeswari Aghoram

**Affiliations:** 1 Department of General Medicine, Indira Gandhi Medical College And Research Institute, Pondicherry, IND; 2 Department of General Medicine, Indira Gandhi Medical College And Research Institute, Pondicherry, IND; 3 Department of Neurology, Jawahalal Institute of Postgraduate Medical Education and Research (JIPMER), Pondicherry, IND

**Keywords:** empty sella syndrome, refractory hypoglycemia, seizures, panhypopituitarism

## Abstract

An empty sella is reported to occur in 5.5%–23.5% of the population and is usually asymptomatic. It can be associated with endocrine disturbances. We report a 48-year-old woman who presented with refractory hypoglycemia, seizures, and shock that improved with levothyroxine, hydrocortisone, and octreotide. Investigations revealed central hypothyroidism, hypoprolactinemia, low gonadotropins, normal C-peptide and a primary empty sella. Case reports of Sheehan syndrome with or without empty sella causing hypoglycemia have been reported occasionally. Our patient had never become pregnant. She had experienced premature menopause and symptoms suggestive of hypothyroidism for many years (without treatment) before her emergency department admission for altered sensorium.

## Introduction

Busch described the condition empty sella syndrome (ESS) in 1951, in which the sella turcica is partially or completely filled with cerebrospinal fluid (CSF), resulting in displacement or flattening of the normal pituitary gland [1]. Empty sella is reported to occur in 5.5%–23.5% of the population [2]. About 25%–50% patients have endocrine abnormalities, most commonly panhypopituitarism, gonadotropin deficiency or diabetes insipidus [1, 3]. Type 2 diabetes mellitus and impaired glucose tolerance are common glucose-related abnormalities in ESS [4]. Herein, we report a case of empty sella syndrome presenting with seizures, refractory hypoglycemia, and shock.

## Case presentation

This 48-year-old female patient was brought by her mother to the emergency department with complaints of being unarousable two hours after her usual waking time. She was diagnosed to have hypothyroidism two years ago but without compliance with treatment. She had attained menopause at 40 years of age. At 22 years, she had separated from her husband within a year of marriage but she refused to elaborate the reasons for her separation. She did not conceive during her marriage or thereafter. She had been living with her parents ever since and there had been frequent altercations regarding her laziness and the need for them to support her. Her brother had diabetes that was controlled with oral hypoglycemic agents. There was no history of other comorbid illnesses. On examination, she was drowsy, with a bite-mark on the tongue laterally and did not have any focal neurological deficits. Periorbital puffiness, ichthyotic skin, and hoarse voice were observed. Her capillary blood sugar was 24 mg/dl. She was immediately administered 100 mL of an intravenous bolus of 25% dextrose, followed by continuous infusion of 10% dextrose (50g in 2 hours) without significant improvement in either sensorium or capillary glucose levels, which remained <50 mg/dL for two hours. Moreover, she developed hypotension (80 mmHg) within an hour of admission. After obtaining samples for thyroid function and cortisol, intravenous hydrocortisone 100mg was administered. Considering her family history and accessibility to drugs, sulfonylurea overdose was also suspected and hence subcutaneous octreotide 50µg was given. Thereafter, her sugars stabilized between 90 and 140 mg/dL.

**Figure 1:**
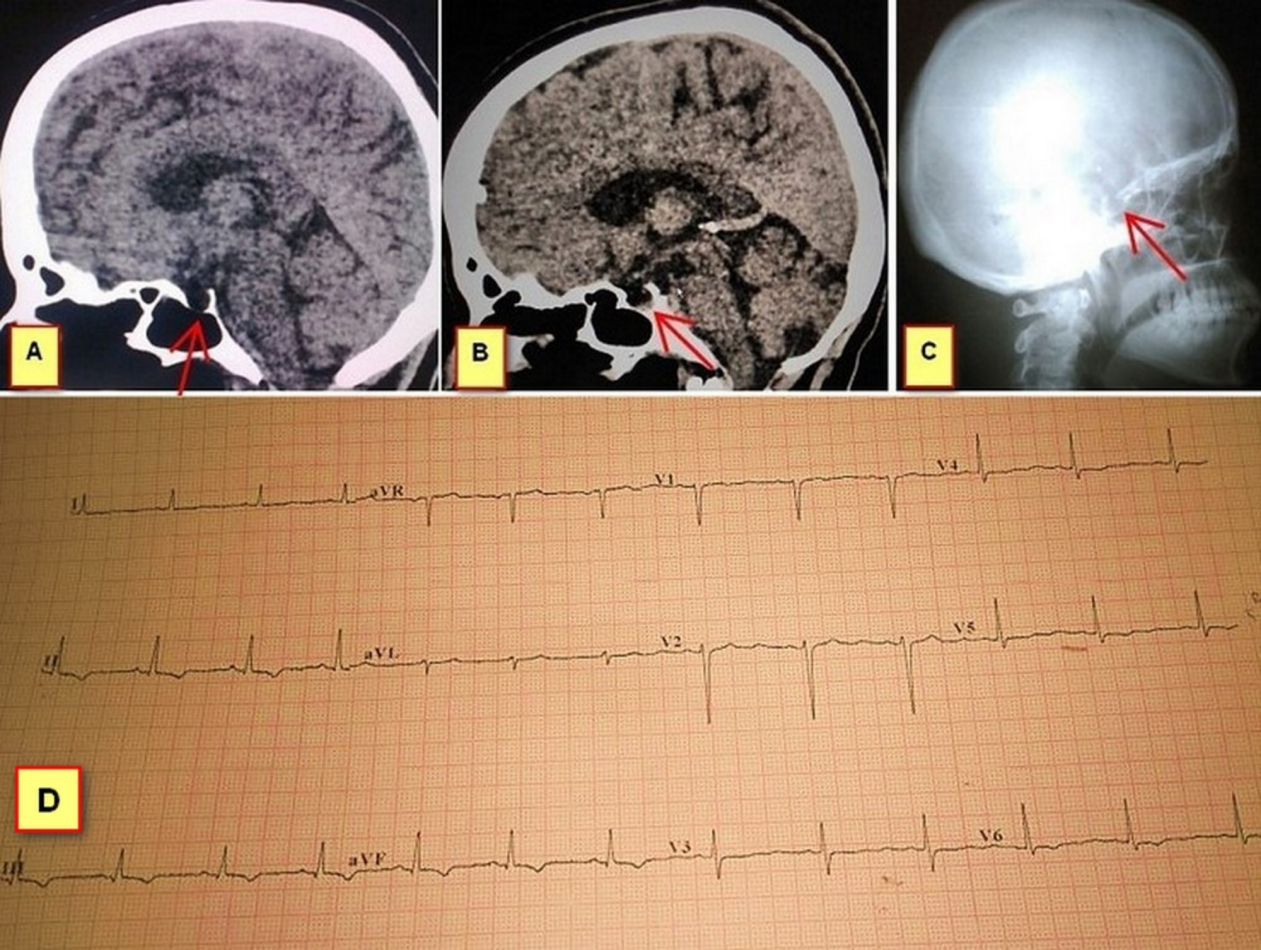
Patient's CT images, skull radiograph, and ECG. A] Sagittal section of CT brain shows an empty sella B] CT brain image of another 45-year-old lady taken from our Radiology department to show a sella with pituitary tissue C] Radiograph of the patient’s skull reveal a deep sella D] ECG of our patient with diffuse T wave inversion in the inferolateral leads

Her initial investigations were as follows: thyroid stimulating hormone (TSH) 1.35 μIU/mL (0.34–4.25), free T4 0.40 ng/dL (0.7–1.24), free T3 1.00 pg/mL (2.4–4.2) and random serum cortisol 12.40 μg/dL (5–25). In view of secondary hypothyroidism, computed tomography brain and other hormones analyses were also performed. An empty sella on computed tomography (CT) and skull radiograph, serum prolactin 1.04 ng/ml (1.9–25), luteinizing hormone (LH) 0.97 mIU/mL (16.0–64.0), follicle stimulating hormone (FSH) 0.37 mIU/mL (18.0–153.0) were seen (Figure 1A and Figure 1C; Figure 1B is a control image taken from another 45-year-old female). Electrocardiogram showed T wave inversion in all leads (Figure 1D). Her renal and liver function tests were normal. Fasting C peptide 1.41 ng/mL (0.81–3.85 with corresponding blood glucose being 84 mg/dL) and CT abdomen were performed to rule out pancreatic causes of hypoglycemia. Viral serologies (HIV, hepatitis B, and C) were negative. MRI brain and testing for growth hormone, adrenocorticotrophic hormone (ACTH), and antidiuretic hormone was unavailable in our institution. Following a diagnosis of ESS with hypopituitarism, oral prednisolone 7.5 mg/day and levothyroxine 100 mcg were continued. On a follow-up visit two months later, she was cheerful and did not have a recurrence of symptoms.

## Discussion

Empty sella syndrome is pathophysiologically characterized by either anatomic abnormalities in the diaphragma sellae (primary ESS) or damage to the pituitary by irradiation/surgery, or autoimmunity leading to the availability of “empty” space in the sella (secondary ESS) [1]. Empty sella has female preponderance, and primary ESS had been commonly described in middle-aged obese women [4]. Empty sella is generally asymptomatic and may be incidentally detected during neuroimaging. Neurological dysfunction such as headache and visual abnormalities and endocrine dysfunction related to prolactin, glucose metabolism, gonads, thyroid glands and the posterior pituitary is observed.

Hypoglycemia is an important cause of altered sensorium in the emergency department. Severe hypoglycemia in the emergency department arises due to drugs, infections, tumors and autoimmune diseases [5]. Our patient presented with seizure (as evidenced by tongue bite) following refractory hypoglycemia that did not improve with intravenous dextrose, necessitating the use of subcutaneous octreotide. Definitions for refractory hypoglycemia are unclear. Most reported cases describe “refractory” either as prolonged duration of hypoglycemia (hours to days), high dose of dextrose (> 30g glucose) to normalize sugar levels or prolonged infusion of glucose to maintain euglycemia [6]. Hypoglycemia may be either hyperinsulinemic (e.g., insulinoma and early diabetes) or hypoinsulinemic (e.g., alcohol ingestion and cortisol deficiency) [7]. Our patient’s normal C-peptide and CT abdomen favored hypoinsulinemic hypoglycemia due to hormone deficiencies (cortisol, growth hormone, glucagon, thyroid and parathyroid hormone), malnutrition, alcohol ingestion, liver failure and non-islet tumors (colorectal, multiple myeloma and lymphoma) [7-8]. Depending upon the cause refractory hypoglycemia may require steroids, octreotide or even cancer chemotherapy [9]. Refractory hypoglycemia has been reported as the presenting manifestation of umbilical artery catheterization, oral hypoglycemic drug use/poisoning, and tumors [8, 10-11]. Rarely, glycogen storage disorders (Ia, Ib and III) can present in adulthood with fasting hypoglycemia and hypothyroidism (autoimmune, in Ib) [12].

There are some reports of Sheehan syndrome presenting with hypoglycemia due to panhypopituitarism [1314]. Some of these patients have had a partial or an empty sella. There is one report of spontaneous hypoglycemia with seizures and coma in a pregnant woman with hypopituitarism (without empty sella), and whose symptoms improved with cortisol replacement therapy [15]. Similar to our case, Bala et al. described a young woman who presented with hypoglycemic shock but was found to have adrenocorticotropin deficiency, empty sella syndrome, and history to suggest Sheehan syndrome [16]. Our patient had never conceived children and did not have any history of acute events like headache, ocular problems or bleeding diathesis in the past, to consider pituitary necrosis.

## Conclusions

Hypoglycemia due to both acute and chronic illnesses is a common presentation to the emergency department. In patients with refractory hypoglycemia that cause seizures and coma, the treating physician needs to focus on drug overdose, malignancies, and endocrinopathies involving the pancreas, thyroid (hypothyroidism) and pituitary glands. Detailed history, appropriate imaging and hormonal analyses and replacement would be necessary to manage such patients.
